# Stratifin (SFN) Regulates Cervical Cancer Cell Proliferation, Apoptosis, and Cytoskeletal Remodeling and Metastasis Progression Through LIMK2/Cofilin Signaling

**DOI:** 10.1007/s12033-023-00946-1

**Published:** 2023-11-09

**Authors:** Naiyi Du, Daojuan Li, Wei Zhao, Yakun Liu

**Affiliations:** 1 Department of Gynecology, The Fourth Hospital of Hebei Medical University, No. 12 Jiankang Road, Shijiazhuang, 050011 Hebei Province China; 2 Cancer Institute, The Fourth Hospital of Hebei Medical University, Shijiazhuang, 050011 Hebei Province China

**Keywords:** Stratifin, Cervical cancer, LIMK2, Cofilin, Cytoskeletal

## Abstract

The aberrant expression of Stratifin (SFN) is intricately associated with the initiation and progression of numerous tumors. This study aims to investigate whether SFN regulates the metastasis of cervical cancer cells through the LIMK2/Cofilin signaling pathway. In this study, we compared the expression of SFN in normal cervical tissues and cervical carcinoma tissues. We established SFN overexpression and SFN silencing cellular models to assess the invasive and migratory capabilities of cervical cancer cells using transwell and scratch assays. YO-PRO-1/PI and EdU staining were employed to evaluate apoptotic and proliferative capacities, while Actin-Tracker Green-488 was utilized to investigate cytoskeletal remodeling. The expression levels of SFN, LIMK2, p-LIMK2, Cofilin, and p-Cofilin were examined through Western blotting and immunofluorescence. Our findings revealed elevated expression of SFN in cervical squamous cell carcinoma tissues. SFN overexpression was observed to enhance invasion and migration of cervical cancer cells, induce cytoskeletal remodeling, facilitate cell proliferation, and suppress apoptosis. Furthermore, SFN overexpression upregulated the expression levels of LIMK2, p-LIMK2, Cofilin, and p-Cofilin. Conversely, silencing SFN exerted opposite effects. SFN plays an important role in the diagnosis of cervical cancer. SFN can regulate cervical cancer cell proliferation, apoptosis, cytoskeletal remodeling and metastasis through LIMK2/Cofilin signaling.

**Supplementary Information:**

The online version contains supplementary material available at 10.1007/s12033-023-00946-1.

## Introduction

Cervical cancer, a prevalent malignancy among women, primarily arises from infection of human papillomavirus (HPV) [1]. However, not all HPV-infected women progress to cancer. Research suggests that additional auxiliary factors are involved in the transition from viral infection to cervical cancer, wherein certain pivotal auxiliary factors play a role in the advancement of the disease [2]. Therefore, unraveling the key factors in the progression of cervical cancer at the molecular biology level is crucial for understanding its pathogenesis.

14-3-3 proteins constitute a widely distributed family of proteins presented in eukaryotic cells, including seven subtypes (β, γ, ε, η, σ, τ, ζ) [3]. Among them, 14-3-3σ protein, also known as Stratifin (SFN), exhibits the closest association with tumors, being frequently downregulated or absent in most malignancies [4]. SFN protein plays a pivotal role in DNA damage repair and regulation of the cell cycle [5]. In cancer research, SFN exhibits a "double-edged sword" effect, as it can also inhibit the invasion and metastasis of tumor cells [6, 7]. However, in colorectal cancer [8], esophageal cancer [9], and other malignancies, SFN is found to be highly expressed. SFN is considered a tumor suppressor in approximately 80% of breast tumors, and its expression in normal mammary epithelial cells has been found to be associated with malignant phenotypes in two basal-like breast cancer progression models [10]. SFN guides the invasion of breast tumors by integrating the dynamics of the cellular cytoskeleton, stabilizing soluble actin-myosin complexes and intermediate filaments [10]. In oral squamous cell carcinoma, SFN is overexpressed in cancer tissues and is associated with reduced overall survival, serving as an adverse prognostic indicator for oral squamous cell carcinoma [11]. In cervical cancer research, the expression of SFN in squamous cell carcinoma is significantly higher compared to adenocarcinoma [12]. Importantly, the molecular mechanisms underlying the role of SFN in the progression of cervical squamous cell carcinoma remain unclear.

Members of the LIM kinase (LIMK) family, including LIMK1 and LIMK2, are serine protein kinases involved in regulating actin polymerization and microtubule depolymerization. The actin-severing protein, Cofilin, serves as its primary effector. The interplay between LIMK/Cofilin signaling and cell cytoskeletal remodeling plays a crucial role in tumor cell invasion and metastasis [13, 14]. The LIMK/Cofilin signaling pathway is closely associated with the clinical pathological features of gastric cancer, such as depth of invasion and distant metastasis. Compared to adjacent normal gastric tissue, gastric cancer tissue exhibits upregulated expression of LIMK1, LIMK2, and Cofilin-1, while phosphorylated Cofilin-1 expression is downregulated [15]. The RhoA-LIMK2-Cofilin-1 signaling pathway regulates the organization of the actin cytoskeleton, affecting cell adhesion and promoting morphological changes and metastasis in colorectal cancer cells [16].

During the process of cancer cell morphological transformation, the interaction between SFN and LIMK2-cofilin signaling pathway plays a role by influencing cellular cytoskeletal remodeling. However, the precise relationship between SFN and LIMK2-cofilin remains unclear. Based on the aforementioned studies, the objective of this investigation is to explore whether SFN regulates the proliferation, apoptosis, cytoskeletal remodeling and metastasis of cervical cancer cells through the LIMK2-cofilin signaling pathway.

## Materials and Methods

### Cell Culture and Transfection

Both Human cervical carcinoma cell lines Caski and SiHa (Shanghai Zhong Qiao Xin Zhou Biotechnology Co.,Ltd., https://www.zqxzbio.com/) were cultured in dulbecco's modified eagle medium (DMEM; Corning, USA) supplemented with 10% fetal bovine serum (FBS; Corning), 1% penicillin along with streptomycin at 37℃ and 5% CO_2_. SFN-small interfering RNA (SFN-siRNA) (Sense UCUCAGUAGCCUAUAAGAACGUGGU, Antisense ACCACGUUCUUAUAGGCUACUGAGA), pcDNA 3.1, and SFN overexpressing plasmid (SFN-OE) were purchased from Sangon Biotech (Shanghai) Co., Ltd.. Cell transfection were carried out according to the manufacturer's protocol of lipo8000™ transfection reagent (Beyotime, China).

### Human Cervical Cancer Tissue Specimens

We included 67 case group specimens and these were confirmed pathologically to be cervical squamous cell carcinoma. We obtained 67 control specimens from patients who had undergone hysterectomy for benign uterine diseases and had no cervical lesions as confirmed by pathology. The 67 patients from whom the samples were obtained had undergone radical hysterectomy without previous radiotherapy and chemotherapy in 2021.

### Immunohistochemistry (IHC) Staining

For both Normal cervical tissue and cervical carcinoma tissue were first fixed in 10% neutral formalin and embedded in paraffin. After dewaxing and antigen repair, paraffin sections were sealed with goat serum for 30 min. Then the primary antibodies SFN (1:100, 66,251–1-lg, Proteintech, China) was used to incubated the paraffin sections overnight at 4 ℃. The next day, sections were stained with HRP-conjugated secondary antibodies (PV-9000; ZSGB-BIO, China) and DAB, dyed with hematoxylin, sealed with neutral resin. Microscope (Olympus, BX61) was used for observation and photograph [17].

### Transwell Migration Assay

Cells that had been starving for 24 h were resuspended with serum-free medium until the cell concentration is 1 × 10^5^ cells /ml. Added 200 μl cell suspension to the transwell upper chamber and 600 μl medium supplemented with 10% FBS to the lower chamber. After 48 h cultivating, cells were fixed with 4% paraformaldehyde and stained with 0.1% crystal violet. Cotton swab was used to remove the cells remain in the upper chamber, cells migrated to the lower chamber were counted to determine the invasion and migration capabilities of cells.

### Wound-Healing Scratch Assay

5 × 10^5^ cells/well were inoculated onto a six well plate and cultured until the cell monolayer attached to it. 200 μl pipetting tips were used to scratched two parallel lines in six well plate. Serum-free medium was used to continue cells culturing at 37℃, 5% CO_2_ for 48 h. Quantization of the wound-closing process was determined by the gap between the lines at 0 h and 48 h under an inverted optical microscope.

### Apoptosis Detection by YO-PRO-1/PI Staining

According to the Apoptosis and Necrosis Detection Kit with YO-PRO-1 and PI (C1075S, Beyotime Institute of Biotechnology, China) instruction, after washing the adherent cells with PBS, YP1/PI detection working solution was added to the six-hole plate. Cells were then incubated at 37℃ in dark for 20 min. After incubation, the results can be observed under a fluorescence microscope (200 × , Olympus, Japan).

### EdU Proliferation Assay

BeyoClick™ EdU Cell Proliferation Kit with Alexa Fluor 594 (C0078S, Beyotime Institute of Biotechnology, China) was used to detect the cell proliferation of SFN overexpressed or SFN knocked down Caski and SiHa cells. Overnight cultured cells were washed with PBS and incubated with 10 μM EdU for 2 h at 37℃, 5% CO_2_. Then cells were fixed with 4% paraformaldehyde for 15 min and incubated at room temperature with PBS containing 0.3% Triton X-100 for 15 min. After washed with PBS, cells were incubated with click reaction at room temperature in dark for 30 min, until which the number of EdU positive cells can be observed through a fluorescence microscope (200 × , Olympus, Japan).

### Immunofluorescent Staining

First, Cells were fixed with 4% paraformaldehyde at room temperature for 10 min. Then 0.5% Triton X-100 was used to permeabilize cells at 37℃ for 15 min. Cells were incubated with goat serum at 37℃ for 30 min to block non-specific binding proteins. After washed with PBS for three times, cells were incubated with the following primary antibodies: SFN(1:200, 66251-1-lg, proteintech, China), LIMK2(1:200, 12350-1-AP, proteintech, China), Cofilin(1:200, 10960-1-AP, proteintech, China), p-Cofilin(1:200, AF3232, Affinity, USA) at 4℃ overnight. The second day, cells were incubated with Alexa Fluor® 488 Conjugate (1:100; ZF-0512; ZSGB-BIO, China) or Alexa Fluor® 594 Conjugate (1:100; ZF-0513; ZSGB-BIO, China) secondary antibodies at 37˚C for 1 h following stained with DAPI until it is dry. Images were captured using a fluorescence microscope (400 × , Olympus, Japan).

### F-Actin Staining

After cultured on glass slides for 24 h, cells were fixed with PBS containing 3.7% formaldehyde at room temperature for 20 min. Diluted Actin-Tracker Green-488 (Beyotime, China) and Alexa Fluor® 594 Conjugate (1:100; ZF-0513; ZSGB-BIO, China) secondary antibody with Immunofluorescence secondary antibody diluent (P0108, Beyotime, China) in a 1:100 ratio for incubating cells in dark at room temperature for 60 min. Cells were washed with PBS, air dried, and sealed with DAPI. Photographs were then taken using a fluorescence microscope (400 × , Olympus, Japan).

### Cell Viability Assay-Cell Counting Kit-8 (CCK8)

Added 100 μl cell suspension into each well of a 96-well plate and cultured cells at 37℃, 5% CO_2_ for 24 h. 10 μl different concentration (5 μM, 10 μM, 20 μM, 40 μM, 100 μM, 200 μM, 300 μM) of TH-257 were added to each well. After 12 h of incubation at 37℃, 10 μl CCK-8 solution was added to each well and cells were continue cultivated for 4 h. OD values were detected using a Microplate reader (Rayto, USA) under 450 nm.

### Western Blotting

The lysis of Caski and SiHa was carried out utilizing RIPA lysis buffer (KeyGEN BioTECH, China), and the protein concentration was detected via BCA assay kit (Leagene Biotechnology, China). 20 μg protein was separated using 11% SDS-PSGE and was transferred onto PVDF membranes. Then blocked the PVDF membranes with 5% skimmed milk dissolved in TBST for non-specific binding for 1 h and incubated the membranes at 4℃ overnight with the following antibodies: SFN (1:7000, 66251-1-lg; proteintech; China); LIMK2 (1:600, 12350-1-AP, proteintech, China); p-LIMK2 (1:1000, AF3346, Affinity, USA); Cofilin (1:1000, 10960-1-AP, proteintech, China); p-Cofilin (1:1000, AF3232, Affinity, USA) and β-actin (1:2000, 20536-1-AP, proteintech, China). In the following day, membranes were incubated with horseradish peroxidase (HRP)-conjugated secondary antibodies:(1:5000, ZB-2301, ZSGB-BIO, China; 1:5000, ZB-2305, ZSGB-BIO, China) for 1 h at room temperature and washed thrice with TBST for protein detection via Super ECL Plus (APPLYGEN, China) [18].

### Statistical Analysis

SPSS version 22.0 (IBM Corp) was used to perform all statistical analysis. Data are represented by mean ± SD. Differences between two samples were compared using T-test, differences between multiple samples were compared using one-way ANOVA. P < 0.05 indicates a significant difference.

## Results

### The Expression of SFN in Cervical Cancer and Its Role in the Metastasis of Cervical Cancer Cells

**Fig. 1:**
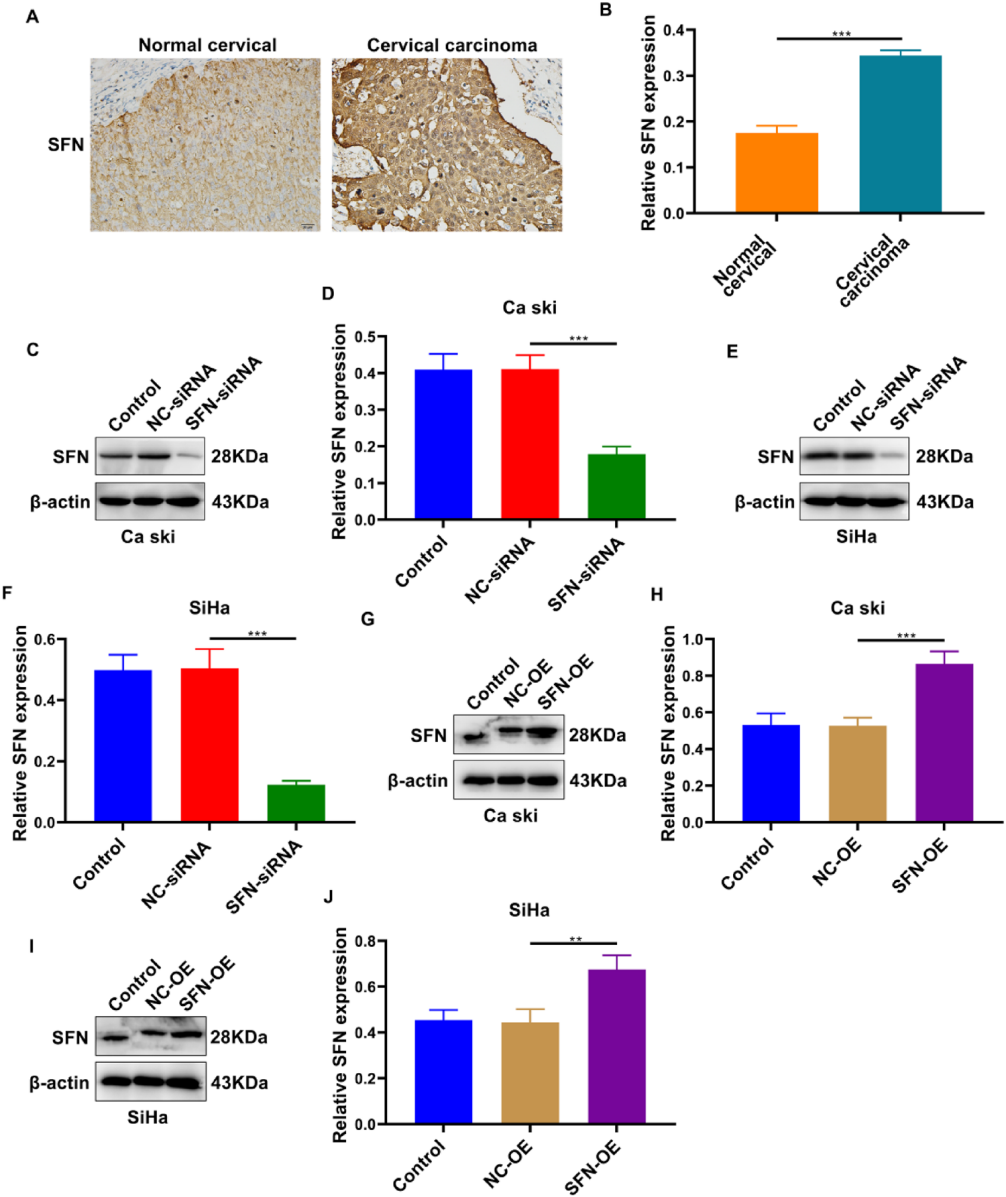
Expression of SFN in cervical carcinoma tissues. **A**–**B** Immunohistochemistry assay results showed that SFN presents high expression in cervical carcinoma tissues than normal cervical tissues. (***p < 0.001, Scale bar, 20 μm, 400 ×). **C**–**J** Both SFN-siRNA and SFN-OE plasmids were successfully transfected into Ca Ski and SiHa cell lines. Western blotting was used to detect the expression level of SFN after transfection. (**p < 0.01; ***p < 0.001)

**Fig. 2:**
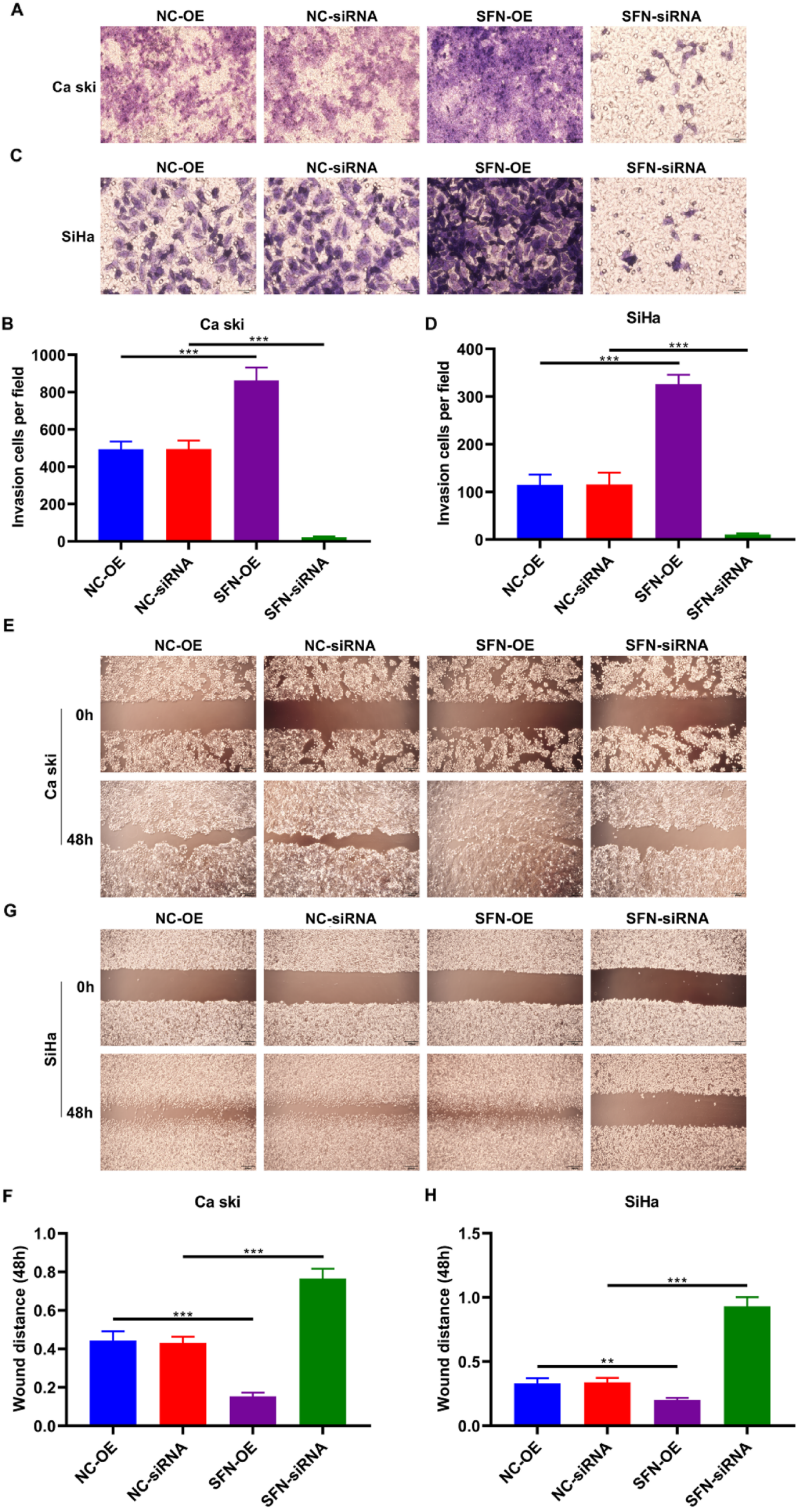
SFN-OE plasmids and SFN-siRNA facilitated and inhibited the invasion and migration respectively both in Ca Ski and SiHa cell lines. **A**–**D** After transfected with SFN-OE plasmids and SFN-siRNA, cell invasion quantity of Ca Ski and SiHa cell lines was detected by transwell assay. (*** *p* < 0.001, Scale bar, 50 μm, 200 ×). **E**–**H** Wound distance of Ca Ski and SiHa cell lines after 48 h culturing. (** *p* < 0.01, *** *p* < 0.001, Scale bar, 200 μm, 40 ×)

Immunohistochemical analysis revealed an upregulation of SFN expression in cervical cancer tissues compared to normal cervical tissues (Fig. 1A–B, Table 1). Western blot experiments demonstrated the successful establishment of SFN-overexpressing (SFN-OE) and SFN-silencing (SFN-siRNA) cellular models for manipulating SFN expression in cervical cancer cells (Fig. 1C–J). Results from Transwell and scratch assays demonstrated that SFN-OE enhanced the invasive and migratory capacities of Caski and SiHa cells, whereas SFN knockdown inhibited the invasion and migration of cervical cancer cells (Fig. 2A–H).

**Table 1:** SFN expression in Normal tissues and Cervical cancer tissues

Groups	N	SFN expression		*P* value
		− (%)	+ (%)	
Normal	67	38 (56.7)	29 (43.3)	0.001
Cancer	67	19 (28.6)	48 (71.4)

### SFN Exerts an Inhibitory Effect on Apoptosis in Cervical Cancer Cells

**Fig. 3:**
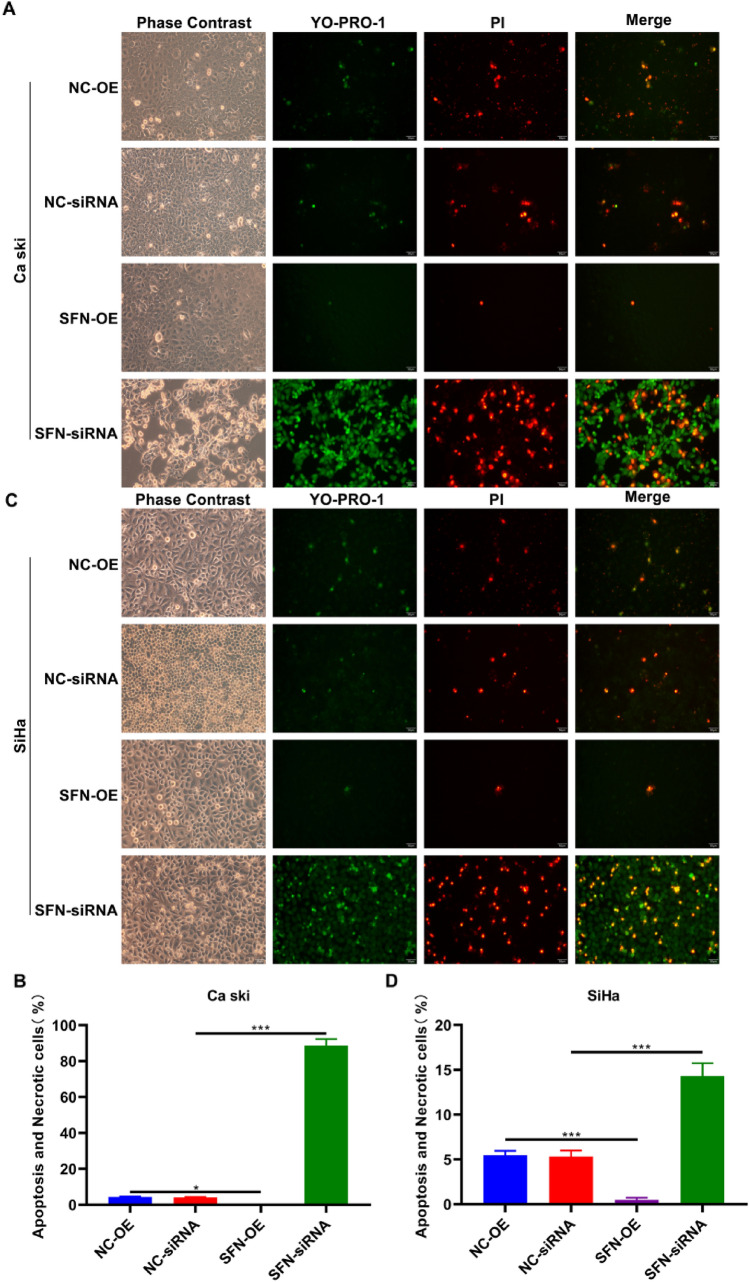
Effect of SFN on the apoptosis of cervical cancer cell. SFN overexpressed and knockdown **A**–**B** Ca Ski cells and **C**–**D** SiHa cells were stained with both YO-PRO-1 and PI dye, apoptotic cells showed green fluorescence, and necrotic cells showed both red and green fluorescence. (** *p* < 0.01, *** *p* < 0.001, Scale bar, 50 μm, 200 ×)

The results of the YO-PRO-1/PI assay revealed a significant decrease percentage of apoptotic and necrotic cell in the SFN-OE cell model compared to the control group, while an evident increase in the proportion of apoptotic and necrotic cells was observed in the SFN-siRNA cell model. These findings indicate that SFN exerts regulatory effects on apoptosis in cervical cancer cells (Fig. 3).

### SFN Enhances the Proliferative Capacity of Cervical Cancer Cells and Orchestrates Remodeling of the Cellular Cytoskeleton

**Fig. 4:**
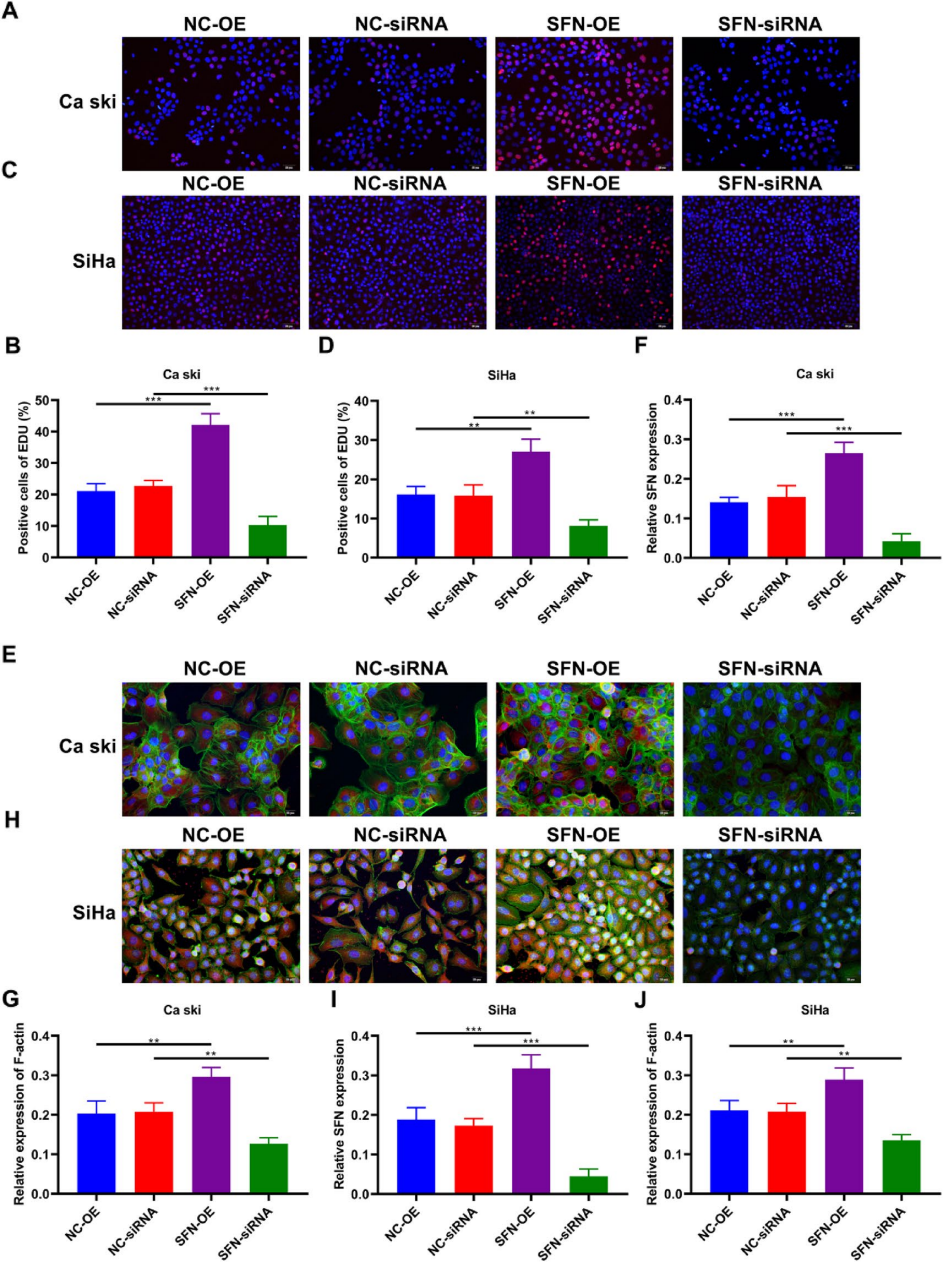
The effect of SFN on the proliferation and remodeling of the cellular cytoskeleton abilities of cervical cancer cells. EdU assay for cell proliferation of **A**–**B** Ca Ski cells and **C**–**D** SiHa cells under transfection of SFN-OE plasmid and SFN-siRNA. Percentage of EdU-positive cells was recorded. **E**–**J** Effect of SFN in Ca Ski and SiHa cell lines on F-actin cytoskeleton. **F**, **I** Immunofluorescent and **G**, **J** Phalloidin staining after SFN-siRNA and SFN-OE plasmids transfection. (** *p* < 0.01, *** *p* < 0.001, Scale bar, 50 μm, 200 × , 20 μm, 400 ×)

The cellular proliferation potential was evaluated using the EdU assay. Our experimental findings unveiled a remarkable phenomenon: the overexpression of SFN (SFN-OE) significantly augmented the population of EdU-labeled cells in both Ca Ski and SiHa cell lines. In contrast, the targeted knockdown of SFN (SFN-siRNA) induced an opposite effect, triggering a discernible decrease in the proportion of EdU-positive cells. These compelling results provide compelling evidence to support the notion that SFN exerts a regulatory influence on the proliferative capacity of cervical cancer cells (Fig. 4A–D). We employed the Actin-Tracker Green-488 assay to visualize and quantify the fluorescence intensity associated with the cellular cytoskeletal framework. Intriguingly, the upregulation of SFN yielded a striking enhancement in the intensity of fluorescent signals. In stark contrast, the depletion of SFN exhibited a notable reduction in fluorescence intensity, underscoring its crucial role in orchestrating cytoskeletal reorganization (Fig. 4E–F).

### SFN Overexpression (SFN-OE) in Cervical Cancer Cells Results in the Upregulation of LIMK2, p-LIMK2, Cofilin, and p-Cofilin

**Fig. 5:**
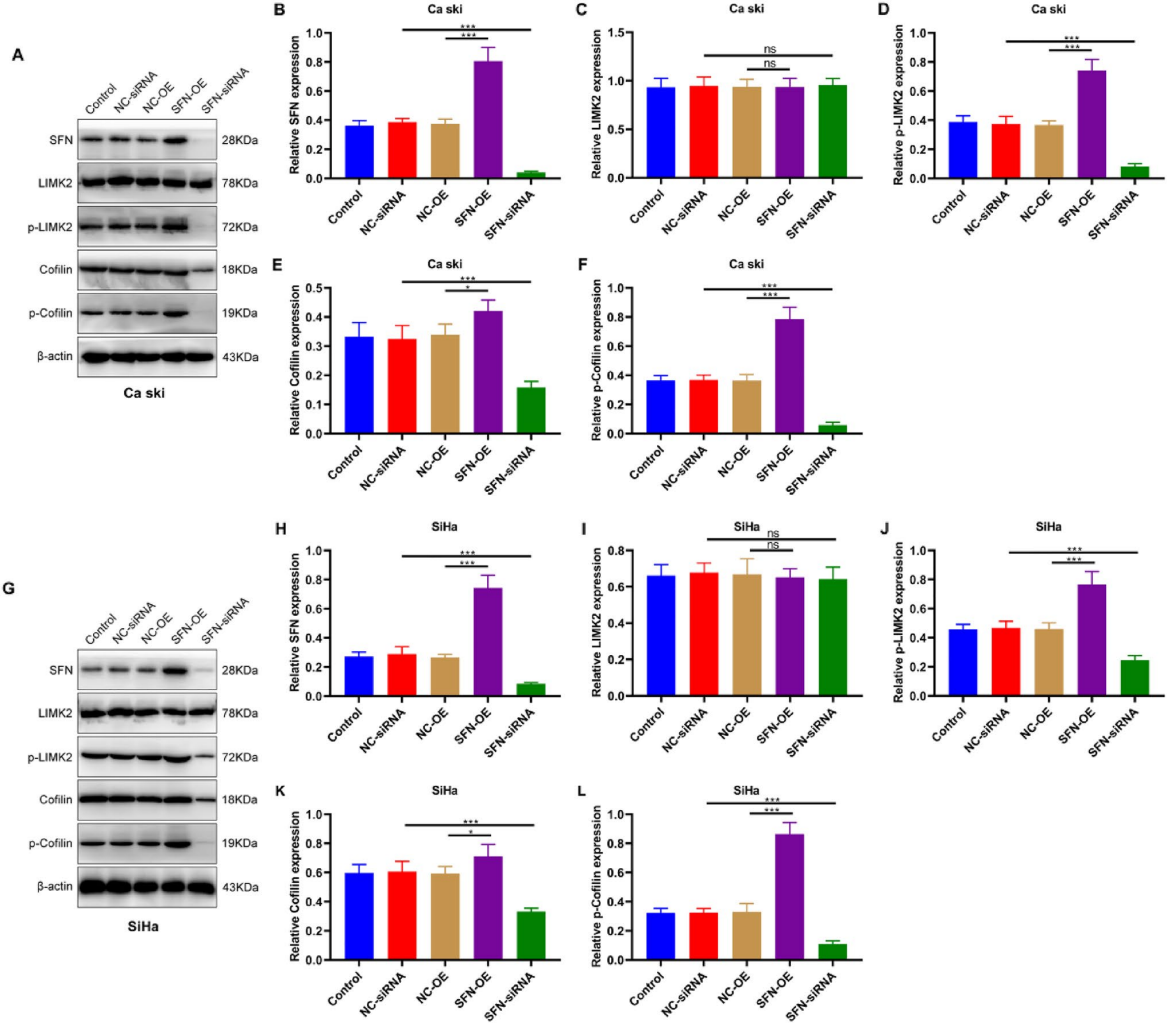
SFN regulates the expression of LIMK2, p-LIMK2, Cofilin, and p-Cofilin in cervical cancer cells. **A** The expression of **B** SFN, **C** LIMK2, **D** p-LIMK2, **E** Cofilin, and **F** p-Cofilin in Ca Ski cells with SFN overexpressed or knocked down were detected by western blotting. **G** The expression of **H** SFN, **I** LIMK2, **J** p-LIMK2, **K** Cofilin, and **L** p-Cofilin in SiHa cells with SFN overexpressed or knocked down were detected by western blotting. (* *p* < 0.05, *** *p* < 0.001, ns: non-significant)

**Fig. 6:**
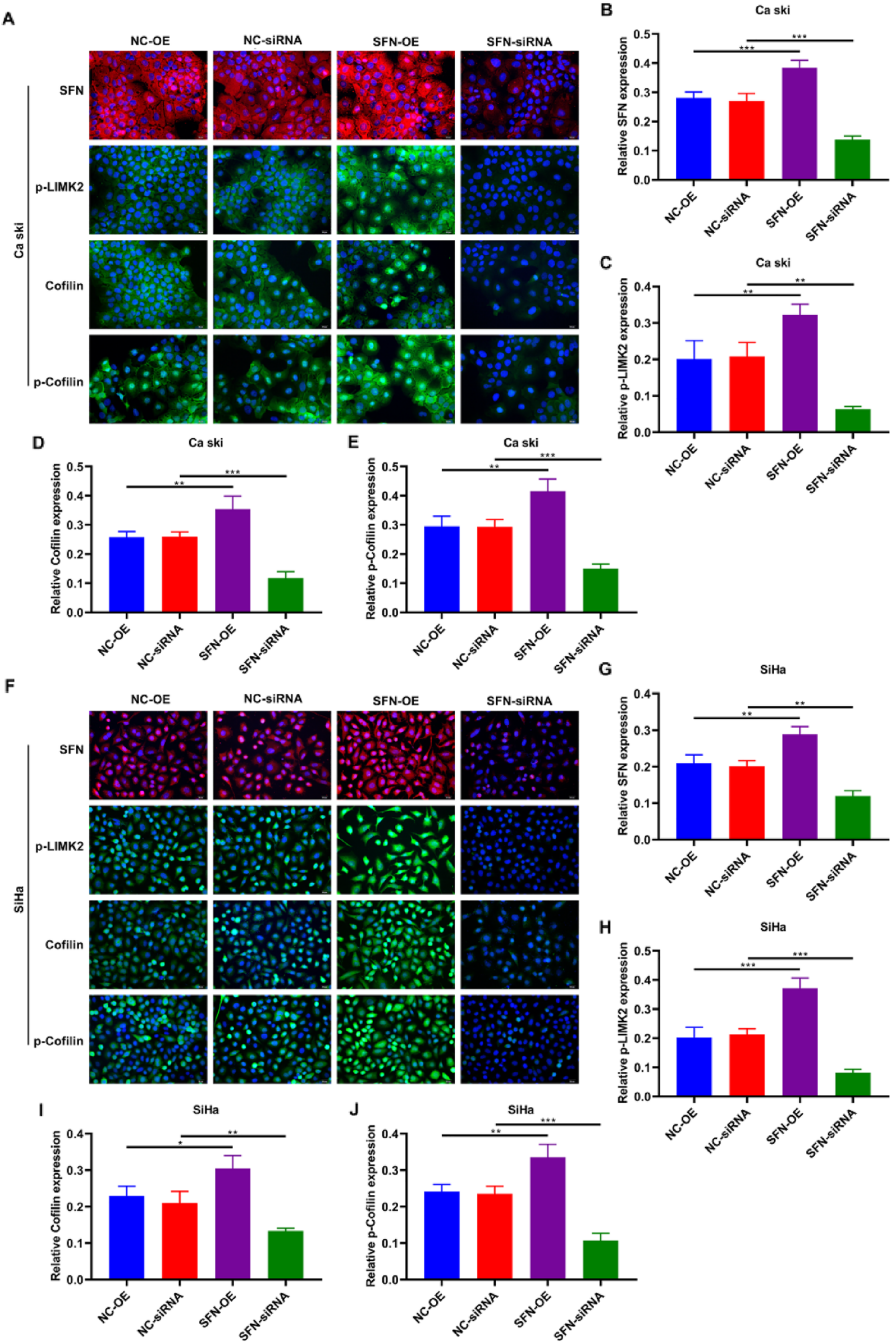
SFN regulates the expression of LIMK2, p-LIMK2, Cofilin, and p-Cofilin in cervical cancer cells. **A** The expression of **B** SFN, **C** p-LIMK2, **D** Cofilin, and **E** p-Cofilin in Ca Ski cells with SFN overexpressed or knocked down were detected by Immunofluorescence analysis. **F** The expression of **G** SFN, **H** p-LIMK2, **I** Cofilin, and **J** p-Cofilin in SiHa cells with SFN overexpressed or knocked down were detected by Immunofluorescence analysis. (** *p* < 0.01, *** *p* < 0.001, Scale bar, 20 μm, 400 ×)

The Western blot analysis revealed distinct effects of SFN overexpression (SFN-OE) and SFN-siRNA on the expression levels of p-LIMK2, Cofilin, and p-Cofilin, while the impact on LIMK2 expression was not statistically significant (Fig. 5). Immunofluorescence staining demonstrated that SFN-OE upregulated the expression of p-LIMK2, Cofilin, and p-Cofilin in both Ca Ski and SiHa cells, whereas SFN-siRNA suppressed their expression (Fig. 6).

### Inhibiting LIMK2 Leads to a Reduction in the Expression Levels of p-LIMK2 and p-Cofilin

**Fig. 7:**
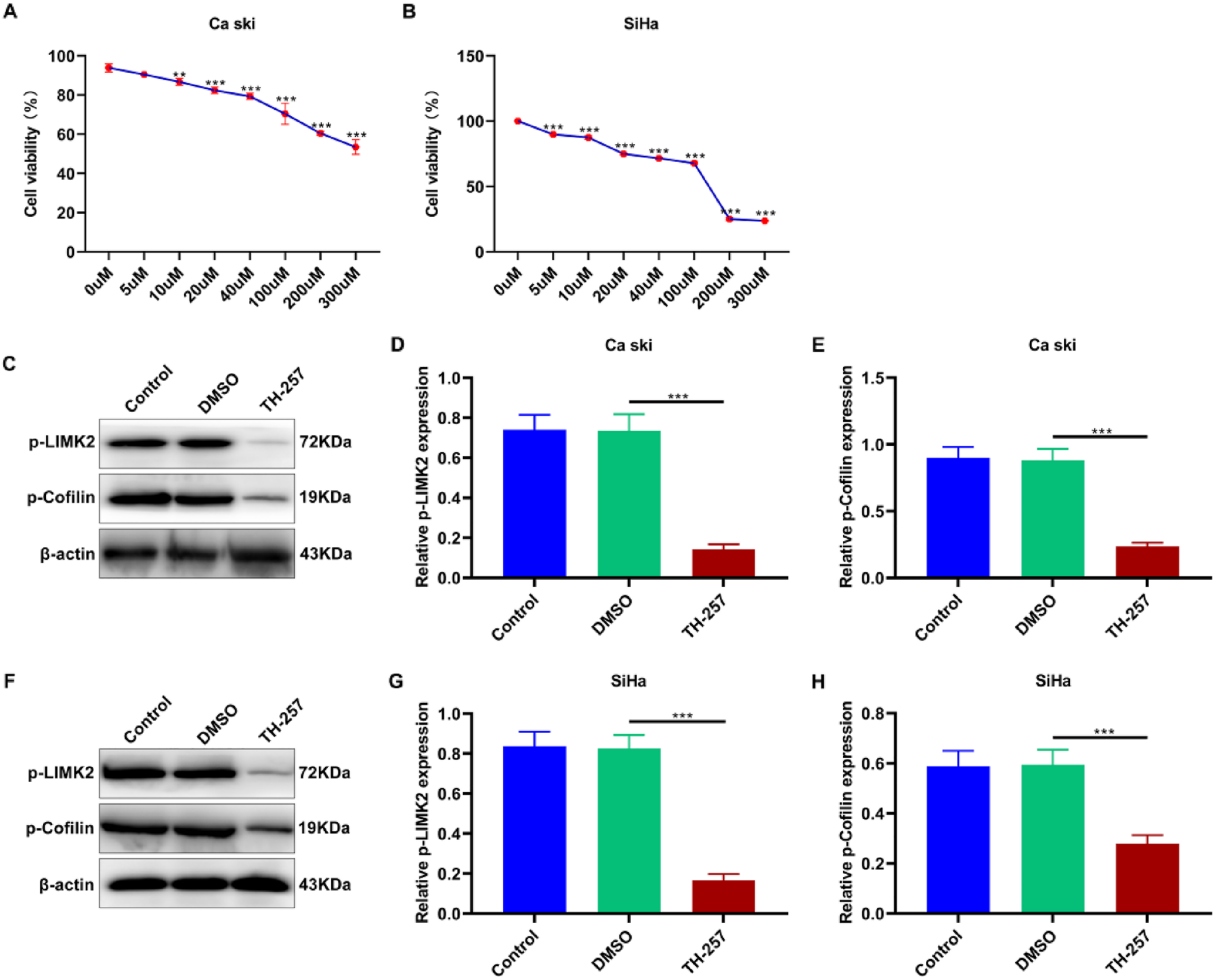
Inhibiting LIMK2 reduced the expression of p-LIMK2 and p-Cofilin. **A**–**B** Effect of different concentrations of an LIMK2 antagonist (TH-257) on the proliferation of Ca Ski and SiHa cells were detected by CCK-8 assay. **C**–**E** The expression of p-LIMK2 and p-Cofilin in Ca Ski cells under TH-257 treatment were detected by western blotting. **F**–**H** The expression of p-LIMK2 and p-Cofilin in SiHacells under TH-257 treatment were detected by western blotting. (** *p* < 0.01, *** *p* < 0.001)

The impact of varying concentrations of TH-257 on the proliferation of two cervical cancer cell lines was assessed through CCK8 experiments. Ultimately, subsequent experiments were conducted using a concentration of 10 μM TH-257 (Fig. 7A–B). From the observations depicted in Fig. 7 C–H, a significant decrease in the expression of p-LIMK2 and p-Cofilin is evident across both cervical cancer cell lines.

## Discussion

Proteomic analysis reveals the involvement of multifactorial interactions in cell cytoskeletal remodeling, cancer cell proliferation, migration, and invasion. SFN exhibits expression in various epithelial tissues, participating in fundamental cellular processes including proliferation, apoptosis, and cytoskeletal integrity [19, 20]. Upon DNA damage, SFN binds to Akt protein, leading to G2/M cell cycle arrest and consequently affecting cancer cell proliferation and apoptosis [21]. In gastric cancer research, a positive correlation between SFN and Ki-67 suggests the crucial role of SFN in gastric cancer cell proliferation [22]. In oral squamous cell carcinoma, both SFN and cytoskeletal proteins are overexpressed and contribute to tumor development [23]. In this study, the expression of SFN in cervical cancer tissues was significantly higher than that in normal tissues. Up-regulation of SFN expression promoted the proliferation, cytoskeletal remodeling, migration and invasion of cervical cancer cells, and enhanced the expression of p-LIMK2, LIMK2, Cofilin and p-Cofilin. SFN knockdown significantly reduced the migration and invasion of cervical cancer cells, down-regulated the expression of p-LIMK2, LIMK2, Cofilin and p-Cofilin, inhibited cytoskeletal remodeling, and increased the apoptosis index of cervical cancer cells. These results indicate the role of SFN in the occurrence and progression of cervical cancer.

Abundant experimental evidence suggests a correlation between aberrant SFN expression and tumor metastasis and invasion. In the study conducted by Zhang et al. [24], miR-675-5p was found to downregulate SFN protein expression, thereby promoting invasion and metastasis in nasopharyngeal carcinoma. The invasion and migration of breast cancer cells may be associated with the upregulation of SFN through silencing of profilin A [25]. Other experiments have also concluded that SFN expression facilitates breast cancer invasion [10]. The results of this study indicate that SFN has important clinical value in the pathological diagnosis of cervical cancer. Importantly, elucidating the mechanism of action of SFN in cervical cancer cell metastasis is the focus of this study. Knockdown of SFN diminished cervical cancer cell metastasis and conversely; hence SFN is more likely a positive regulator of metastasis; it could be considered as a potential biomarker of the progression of cervical cancer. The experimental findings by Boudreau et al. shed light on the mechanistic role of SFN in guiding cell migration and tumor invasion through the regulation of cytoskeletal solubility and dynamics [10]. Within the framework of cytoskeletal remodeling, LIMK2 exerts its influence on cell proliferation by mediating the reshaping of actin cytoskeleton [26]. In numerous tumor studies, it has been established that LIMK2, through phosphorylation of cofilin, modulates actin polymerization, thereby restraining tumor cell invasion [27]. Silencing of LIMK2, by reducing cofilin phosphorylation, disrupts the upregulation of genes associated with epithelial-to-mesenchymal transition (EMT) and impedes cell migration [28]. Our results show that SFN has a significant regulatory effect on the expression of p-LIMK2 and p-Cofilin. Interestingly, SFN can affect cytoskeletal remodeling of cervical cancer, and this effect is closely related to metastasis. Based on these observations, it is reasonable to think that SFN may regulate cervical cytoskeletal remodeling through LIMK2/Cofilin pathway and affect the metastasis of cervical cancer cells. Unfortunately, this study only elaborated the mechanism of SFN regulating cervical cancer cell metastasis at the cellular level. In the future, we will further confirm the mechanism of SFN in cervical cancer progression and tumor immunity using animal models.

Furthermore, our study provides compelling evidence for the pivotal role of LIMK2 in cervical cancer cell metastasis, as demonstrated by the utilization of a potent LIMK2 inhibitor. LIMK2 inhibitor, at therapeutic doses, can represent a therapeutic agent to be further investigated to treat cervical cancer. Remarkably, our findings support the notion that LIMK2 exerts its influence on metastasis through the regulation of Cofilin, aligning with numerous previously reported investigations. Therefore, we speculate that SFN is high likely to regulate the development and progression of cervical cancer cells through LIMK-Cofilin signaling pathway. Nonetheless, the absence of in vivo validation remains a limitation of our study, warranting future investigations to shed light on this aspect.

Elucidating the roles of key protein factors in the process of cervical cancer metastasis holds great promise for the development of improved therapeutic strategies for patients. Through our experimental investigations, it becomes evident that SFN exhibits regulatory control over crucial aspects of cervical cancer cell behavior, encompassing proliferation and apoptosis, invasion, and metastasis. Remarkably, both the overexpression and depletion of SFN have a profound impact on the protein expression levels of LIMK2 and Cofilin. Notably, the SFN and LIMK2/Cofilin signaling pathways converge to actively participate in the remodeling of the cellular cytoskeleton, thus suggesting a plausible mechanism by which SFN orchestrates cervical cancer cell metastasis via modulation of the LIMK2/Cofilin signaling pathway. This pivotal conclusion not only provides a theoretical foundation but also furnishes empirical evidence to support subsequent investigations targeting SFN as a means to decipher the underlying mechanisms and devise therapeutic interventions against cervical cancer cell metastasis. For the apoptosis experiment, we selected YO-PRO-1/PI apoptosis and necrosis detection kit to detect the apoptosis of cervical cancer cells induced by SFN, Its main advantage is to observe apoptosis in different groups under fluorescent labeling microscope directly, false positives caused by pancreatic enzyme digestion and artificial blowing of cells are avoided. Undeniably, flow cytometry is still the best choice for detecting apoptosis [29], in the future, we will combine these two techniques to jointly elucidate the effects of SFN on apoptosis.

To sum up, our work identifies the role of SFN in cervical cancer cell metastasis. SFN can regulate cervical cancer cell proliferation, cytoskeletal remodeling and metastasis through LIMK2/Cofilin signaling. Our results complement the current mechanism of action by which SFN regulates the development and progression of cervical cancer cells through the LIMK2/Cofilin signaling pathway, providing a potential target for cervical cancer treatment.

## Supplementary Information

Below is the link to the electronic supplementary material.

Supplementary file1 (DOCX 994 kb)

## Data Availability

The data that support the findings of this study are available from the corresponding author upon reasonable request.
