# Supplementary material for: Stratifin (SFN) Regulates Cervical Cancer Cell Proliferation, Apoptosis, and Cytoskeletal Remodeling and Metastasis Progression Through LIMK2/Cofilin Signaling
Source: Mol Biotechnol. 2023 Nov 9;66(11):3369–81. doi: 10.1007/s12033-023-00946-1 (PMC11549181; doi:10.1007/s12033-023-00946-1)
Supplement: Supplementary file 1 — Supplementary file1 (DOCX 994 kb) [file 12033_2023_946_MOESM1_ESM.docx]

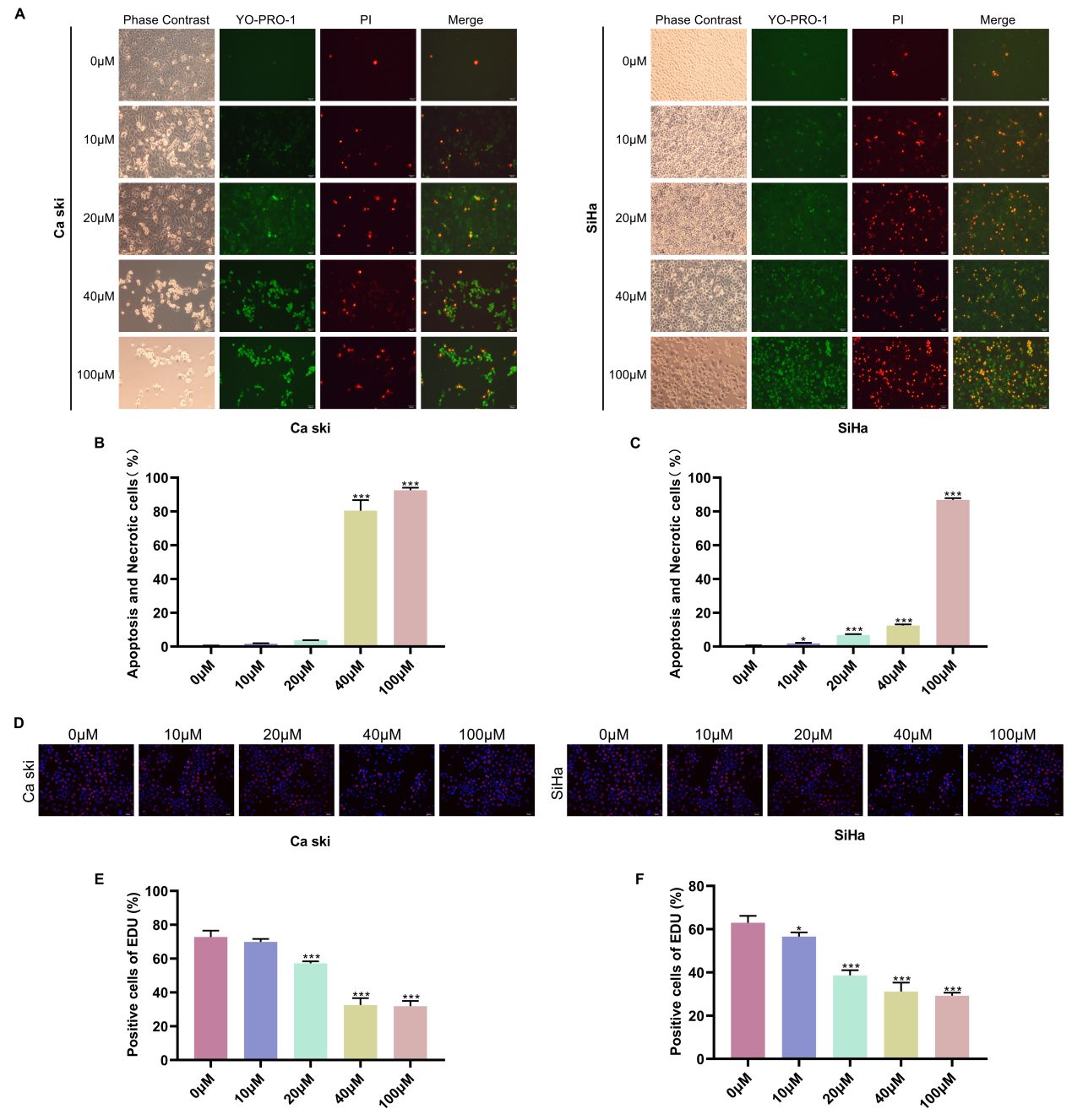


**Supplementary Fig 1** Correlation between the reduction of proliferation and the induction of apoptosis in dose-dependent conditions. Ca Ski and SiHa cells were treated with different concentrations of an LIMK2 antagonist (TH-257). (A-C) Ca Ski cells and SiHa cells were stained with both YO-PRO-1 and PI dye, apoptosis and necrotic cells were counted. (D-F) EdU assay for cell proliferation of Ca Ski cells and SiHa cells, percentage of EdU-positive cells was recorded.( * P<0.05, *** P<0.001, Scale bar, 50 μm, 200×)
